# High expression of *TLR3* in triple-negative breast cancer predicts better prognosis—data from the Fudan University Shanghai Cancer Center cohort and tissue microarrays

**DOI:** 10.1186/s12885-023-10721-9

**Published:** 2023-04-01

**Authors:** Lei Fan, Xin-Yi Sui, Xi Jin, Wen-Juan Zhang, Peng Zhou, Zhi-Ming Shao

**Affiliations:** 1 Department of Breast Surgery, Department of Oncology, Fudan University Shanghai Cancer Center, Shanghai Medical College, Fudan University, Shanghai, 200032 China; 2 Parkway Health, Shanghai, China; 3 Runshangshan Medical Center, Shanghai, China

**Keywords:** Triple-negative breast cancer (TNBC), Toll-like receptor 3 (*TLR3*), Cancer survival, Gene expression

## Abstract

**Introduction:**

We have previously reported that Toll-like receptor 3 (*TLR3*) acts as a suppressor gene for breast cancer initiation and progression. In this study, we evaluated the role of *TLR3* in breast cancer using our original Fudan University Shanghai Cancer Center (FUSCC) datasets and breast cancer tissue microarrays.

**Methods:**

Using FUSCC multiomics datasets on triple- negative breast cancer (TNBC), we compared the mRNA expression of *TLR3* in TNBC tissue and the adjacent normal tissue. A Kaplan–Meier plotter was performed to investigate the expression of *TLR3* on prognosis in the FUSCC TNBC cohort. We performed immunohistochemical staining to analyze TLR3 protein expression in the TNBC tissue microarrays. Furthermore, bioinformatics analysis was performed using the Cancer Genome Atlas (TCGA) data to verify the results of our FUSCC study. The relationship between *TLR3* and clinicopathological features was analyzed with logistic regression and the Wilcoxon signed-rank test. The association between clinical characteristics and overall survival in TCGA patients was assessed using the Kaplan–Meier method and Cox regression analysis. Gene set enrichment analysis (GSEA) was performed to identify signaling pathways that are differentially activated in breast cancer.

**Results:**

The mRNA expression of *TLR3* was lower in TNBC tissue than in the adjacent normal tissue in the FUSCC datasets. The *TLR3* had high expression in immunomodulatory (IM) and mesenchymal-like (MES) subtypes and low expression in luminal androgen receptor (LAR) and basal-like immune-suppressed (BLIS) subtypes. High expression of *TLR3* in TNBC predicted better prognosis in the FUSCC TNBC cohort. Immunohistochemical staining of the tissue microarrays showed that *TLR3* had lower expression in breast cancer tissues than in the adject normal tissues. Furthermore, the *TLR3* expression was positively associated with B cell, CD4 + T cells, CD8 + T cells, neutrophils, macrophages, and myeloid dendritic cells. Bioinformatic analysis using high-throughput RNA-sequencing data from the TCGA demonstrated that the reduced expression of *TLR3* in breast cancer was associated with advanced clinicopathological characteristics, survival time, and poor prognosis.

**Conclusions:**

*TLR3* has low expression in TNBC tissue. High expression of *TLR3* in triple-negative breast cancer predicts better prognosis. *TLR3* expression may be a potential prognostic molecular marker of poor survival in breast cancer.

**Supplementary Information:**

The online version contains supplementary material available at 10.1186/s12885-023-10721-9.

## Introduction

Breast cancer is the most common cancer in American women in addition to skin cancers [1]. The American Cancer Society estimates that approximately 268,600 new cases of invasive breast cancer will be diagnosed in women and approximately 41,760 women will die from breast cancer every year [2]. In China, the incidence of breast cancer is growing rapidly and is approximately more than twice the global incidence rate [1]. Breast cancer is now one of the most common cancers and the sixth leading cause of cancer-related deaths among Chinese women. Approximately 11% of all breast cancers worldwide occur in China [3]. The most aggressive subtype of breast cancer is triple-negative breast cancer (TNBC). TNBC is characterized by a lack of progesterone receptors, estrogen receptors, and no ERBB2 gene expression. TNBC tends to have a higher histologic grade and elevated risk of recurrence and metastasis compared to other subtypes [4].

Immunotherapy is a new strategy for the treatment of TNBC. Humans have two types of immune systems—innate and acquired [5]. Recent studies found that the innate immune system plays a role in preventing relapse in women with breast cancer [6]. The innate immune system, also known as the nonspecific immune system or inborn immunity system, is an important subsystem of the overall immune system that comprises the cells and mechanisms that defend the host from infection by other organisms. Toll-like receptors (TLRs) are a class of proteins that play a key role in the innate immune system. They are single, membrane-spanning, noncatalytic receptors usually expressed on sentinel cells such as macrophages and dendritic cells that recognize structurally conserved molecules derived from microbes [7].

Ten different TLRs (TLR1–TLR10) have been identified in humans. Different TLRs recognize their specific ligands. *TLR3* is activated by double-stranded RNA or polyinosinic–polycytidylic acid (poly [I:C]). *TLR3* is expressed not only in immune system cells, such as dendritic cells and macrophages, but also in other cells, such as epithelial, fibroblast, and breast cancer cells [8].

Toll-like receptor 3 (TLR3) is a member of the toll-like receptor family of pattern recognition receptors of the innate immune system [9]. It recognizes dsRNA and initiates an innate immune response. Previous studies found that TLR3 is a biomarker for the therapeutic efficacy of double-stranded RNA in breast cancer [8]. The activation of TLR3 enhances the anti-tumor effects of chimeric antigen receptor–modified T (CAR-T) cells [10]. Furthermore, in situ activation of TLR3 leads to systemic clinical tumor regression and the potentiation of the PD1 blockade [11]. We found that *TLR3* acts as a suppressor gene in breast cancer initiation and progression in our previous two-stage association studies and functional investigations [12]. Furthermore, our study suggested that *TLR3* is less frequently expressed in breast cancer tissues. However, we only investigated 78 cases in the expression studies, and we did not study the influence of *TLR3* expression on survival rates in patients with triple-negative breast cancer.

The aim of the current study was to evaluate the prognostic value of *TLR3* expression in human breast cancer. First, using the multiomics data of our Fudan University Shanghai Cancer Center (FUSCC) TNBC cohort [13], we investigated the expression of *TLR3* in TNBC tissues, the association of *TLR3* and immune cells, and the influence of *TLR3* expression on survival rates in TNBC. Moreover, we performed immunohistochemical staining to analyze *TLR3* expression in breast cancer tissue microarrays. Furthermore, we utilized the data obtained from the Cancer Genome Atlas (TCGA) to verify the results obtained from our FUSCC cohort.

## Materials and methods

### FUSCC-TNBC study cohort

This study included a cohort from the Fudan University Shanghai Cancer Center TNBC study [13]. The research protocol was approved by the Clinical Research Ethics Committee of the Fudan University Shanghai Cancer Center. Informed consent was obtained from patients. Gene-set variation analysis (GSVA) was utilized to calculate the enrichment score of each pathway in each sample. TNBC was classified into four transcriptome-based subtypes: (1) LAR, (2) immunomodulatory, (3) BLIS, and (4) MES [13]. The Gene expression datasets (GSE118527) were extracted from the Gene Expression Omnibus (GEO) database.

### Survival analysis

Survival curves were constructed using the Kaplan–Meier method and compared with the log-rank test. Univariate and multivariate Cox proportional hazards models were used to explore independent prognostic variables. Age, the number of positive lymph nodes, tumor size, homologous recombination deficiency (HRD) scores, PAM50 subtypes, and TNBC immune subtypes were first analyzed using a univariate Cox proportional hazards model. Then, a multivariate analysis of all significant variables was performed using the Cox proportional hazard regression model.

### Tissue microarrays

The tissue microarray (TMA) chips were obtained from the Shanghai Zhuoli Biotechnology Company Ltd. Tissues were obtained from 90 patients. Formalin-fixed, paraffin-embedded core cylinders were punched and then deposited into a recipient paraffin block. A tissue-arraying instrument (Beecher Instruments®, Silver Spring, MD, USA) was used. Ninety paired sections of breast cancer tissues and matched adjacent tissues were cut and placed on slides.

### Immunohistochemistry

The tissue microarray was incubated at 59 °C for 60 min. After being dewaxed in xylene three times and dehydrated in a series of graded alcohols, the chips were treated with 3% hydrogen peroxide and NaN_3_ to exhaust the endogenous peroxidase activity. The chips were microwaved in 10 mM citrate buffer pH 6.0 to unmask the epitopes. Rabbit polyclonal anti-human TLR3 antibody (Abcam, # ab62566), the primary antibody, was diluted 1:50 in phosphate-buffered saline (PBS). The chips were incubated with diluted primary antibody overnight at 4 °C. After PBS wash, the chips were incubated with anti-rabbit IgG secondary antibody at 37 °C for 35 min. Horseradish peroxidase (HRP) conjugate was then applied to the chips. After washing, the chips were incubated with peroxidase substrate diaminobenzidine for two minutes and counterstained with hematoxylin.

### Data mining and bioinformatics analysis

The clinical information and gene expression data of TNBC patients and normal controls were downloaded from the official TCGA website. In addition, there are thousands of breast cancer cases in the METABRIC database, and we have used these data for our analysis. Boxplots were used to visualize expression differences for discrete variables. The RNA-seq gene expression level 3 HTSeq-Counts data of breast cancer patients with TNBC and clinical data were retained and further analyzed.

A Kaplan–Meier plotter (https://kmplot.com) database, a meta-analysis based discovery, and the validation of an online survival biomarkers tool were used to investigate the survival of TNBC in the GEO, the European Genome-phenome Archive (EGA), the TCGA databases, and the METABRIC database.

### Evaluation of tumor-infiltrating immune cells (TIIC)

The CIBERSORT algorithm (http://cibersort.stanford.edu/) was utilized to calculate the tumor-infiltrating immune cell composition [14]. The TIIC immune cells included B cells, plasma cells, T cells, NK cells, macrophages, monocytes, mast cells, dendritic cells, neutrophils, and eosinophils. Perl (Perl Programming Language, version 5.28.1, http://www.perl.org) was conducted to convert IDs and group samples. The Limma package of R (R Project for Statistical Computing, version 3.5.3, https://www.r-project.org) was used to normalize the gene expression data. The TIIC, *P*-value, root mean square error, and Pearson’s correlation coefficient were quantified for each sample. Furthermore, we applied CIBERSORT to calculate the *P*-value for the deconvolution.

### Gene-set enrichment analysis

GSEA was performed to detect whether an a priori defined set of genes showed statistically significant differential expression between the *TLR3* expression groups to identify potential mechanisms underlying the influence of *TLR3* expression on TCGA. GSEA is a computational method that determines whether an a priori defined set of genes shows statistically significant, concordant differences between two biological states. The expression level of *TLR3* was used as a phenotype label. Gene sets with a normal *P*-value < 0.05 and false discovery rate (FDR) < 0.25 were considered to be significantly enriched.

### Statistical analysis

All statistical analyses were conducted using R (v.3.6.0). The expression of *TLR3* in TNBC patients in the TCGA-BRCA dataset was evaluated. The chi-square test and Fisher’s exact test were applied to identify correlations between *TLR3* mRNA expression and the clinical features of breast cancer. The relationship between clinicopathological features and *TLR3* expression was analyzed with the Wilcoxon signed-rank test and logistic regression. The associations of clinical and pathological characteristics with overall survival in TCGA patients were assessed using Cox regression and the Kaplan–Meier method. The cut-off value of *TLR3* expression was determined by its median value. *P* < 0.05 was considered statistically significant.

## Results

### Low TLR3 expression in TNBC in FUSCC datasets

**Fig. 1:**
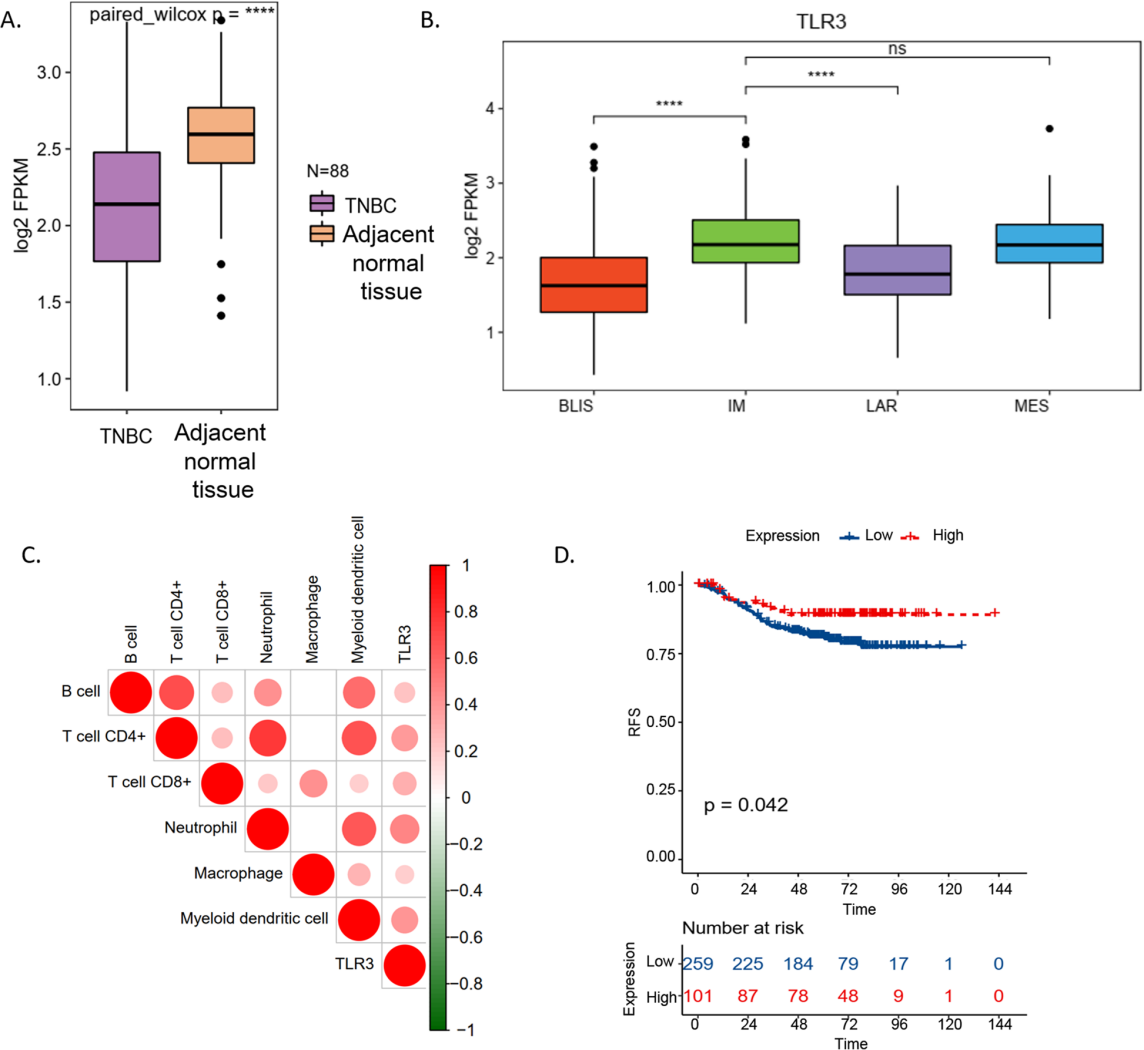
TLR3 and TNBC in FUSCC datasets. The mRNA expression of TLR3 was lower in TNBC tissue than in the adjacent normal tissue (**A**). The TLR3 had high expression in immunomodulatory (IM) and mesenchymal-like (MES) subtypes and low expression in luminal androgen receptor (LAR) and basal-like immune-suppressed (BLIS) subtypes (**B**). The expression of TLR3 was positively associated with B cell, CD4 + T cells, CD8 + T cells, neutrophils, macrophages, and myeloid dendritic cells (**C**). TNBC with high TLR3 expression had a better prognosis than breast cancer with low TLR3 expression (**D**). TLR3: Toll-like receptor 3; FUSCC: Fudan University Shanghai Cancer Center; TNBC: triple-negative breast cancer; IM: immunomodulatory subtype; MES: mesenchymal-like subtype; LAR: low expression in luminal androgen receptor subtype; BLIS: basal-like immune-suppressed subtype; RFS: relapse-free survival

The mRNA expression of *TLR3* was lower in TNBC tissue than in the adjacent normal tissue (Fig. 1A). Furthermore, we investigated the expression of *TLR3* in four TNBC transcriptome-based subtypes. The *TLR3* had high expression in immunomodulatory (IM) and mesenchymal-like (MES) subtypes and low expression in luminal androgen receptor (LAR) and basal-like immune-suppressed (BLIS) subtypes (Fig. 1B). Moreover, the mRNA expression of *TLR3* was positively associated with B cell, CD4 + T cells, CD8 + T cells, neutrophils, macrophages, and myeloid dendritic cells (Fig. 1C).

### High expression of TLR3 in TNBC predicts better prognosis in FUSCC TNBC cohort

A Kaplan–Meier plotter revealed that TNBC with high *TLR3* expression had a better prognosis than breast cancer with low *TLR3* expression (Fig. 1D, *p* = 0.042) in the FUSCC TNBC cohort.

### Microarray immunohistochemistry showed breast cancer tissue had low TLR3 protein expression

**Fig. 2:**
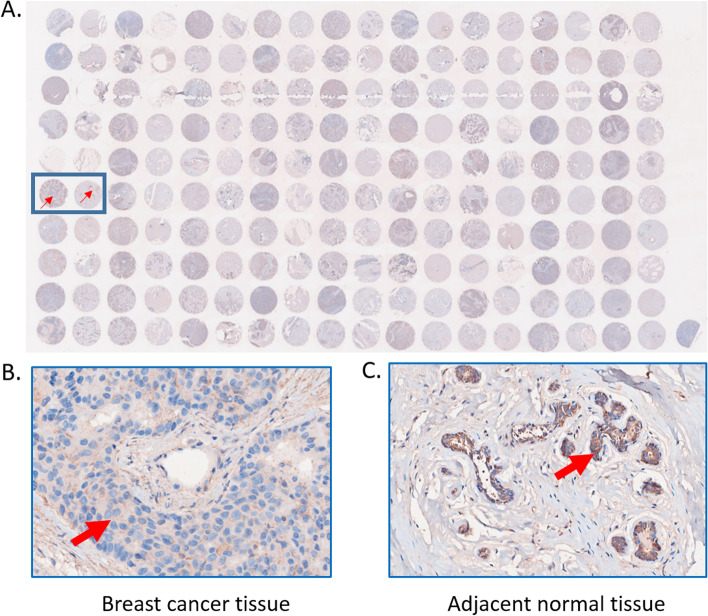
Breast cancer microarray immunohistochemistry. The scan of breast cancer microarray after TLR3 immunohistochemistry staining (**A**). A classic pair of samples (**B** and **C**). Left sample is breast cancer tissue. Right sample is adjacent normal tissue. Breast cancer tissue has higher TLR3 positive staining than adjacent normal tissue

The expression level and the positive rates of TLR3 were compared between breast cancer tissue and adjacent normal tissue samples by immunohistochemistry on microarray. The samples in odd columns are from breast cancer tissues. The samples in even columns are from adjacent normal tissues (Fig. 2A). Figure 2B and C show a classic pair of samples. Breast cancer tissue has lower positive staining than the adjacent tissue. The expression of *TLR3* is lower in the TNBC tissue than the adjacent normal tissue.

### TLR3 is a protective factor of TNBC in online public databases

**Fig. 3:**
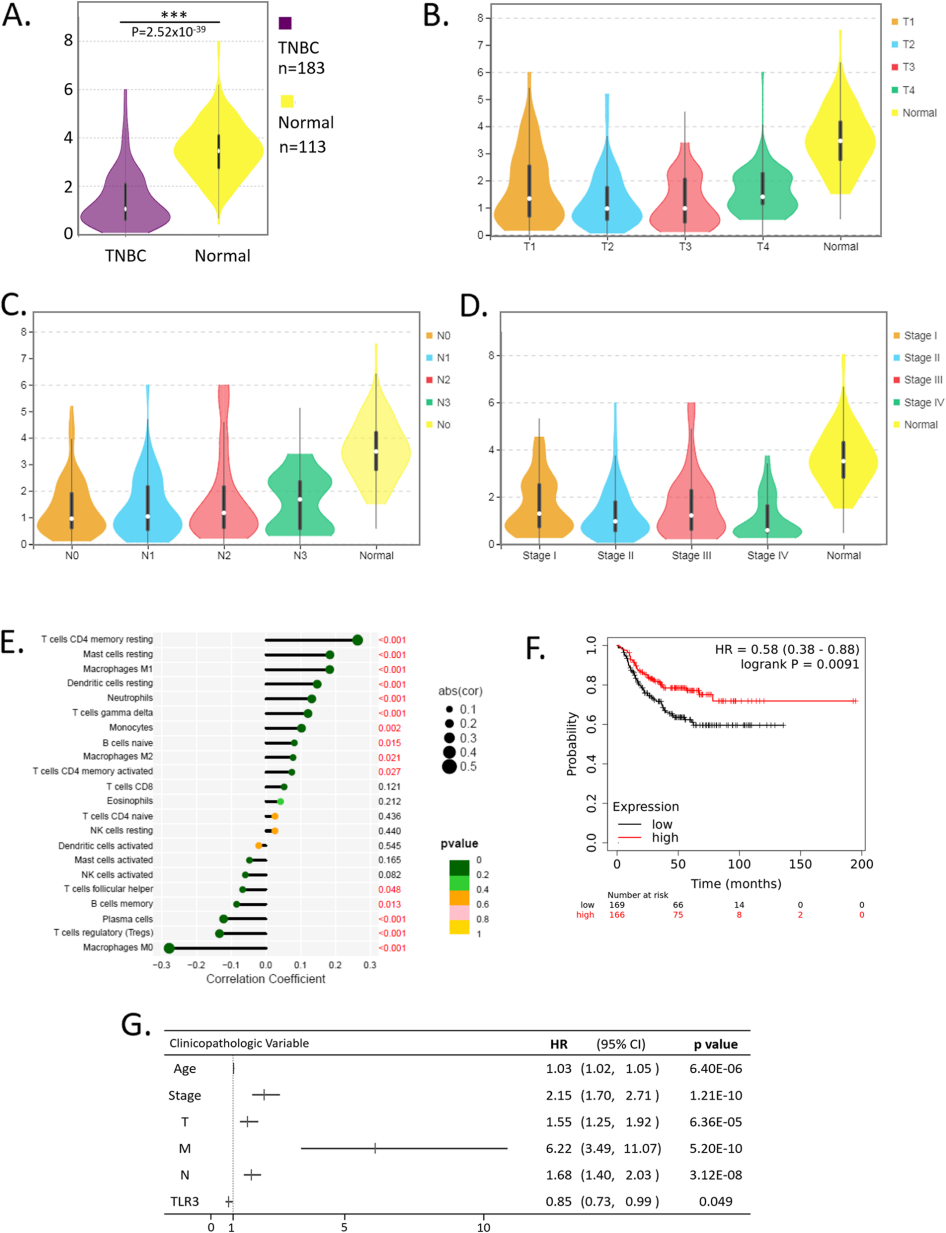
Association of TLR3 expression with clinicopathological characteristics in TCGA cohort. TLR3 expression was lower in the TNBC than in the normal controls (**A**). TLR3 expression in TNBC with different clinicopathological characteristics (**B**, **C**, and **D**). The bar plot visualized the proportions of infiltrating immune cells (**E**). Kaplan–Meier plotter revealed that TNBC with high TLR3 expression had a better prognosis than breast cancer with low TLR3 expression (**F**). Associations of overall survival with clinicopathological characteristics in TCGA patients using Cox regression (**G**). TNBC: triple-negative breast cancer; TLR3: Toll-like receptor 3; T: Tumor of the Tumor, Node, Metastasis (TNM) staging system. N: Node of the Tumor, Node, Metastasis (TNM) staging system. M: Metastasis of the Tumor, Node, Metastasis (TNM) staging system. HR: hazard ratio; CI: 95% confidence interval

First, we found that TNBC had low *TLR3* expression in the TCGA database. In this study cohort, 183 triple-negative breast cancer samples with both clinical and gene expression data were downloaded from the TCGA database. *TLR3* expression data across all patient characteristics were analyzed. *TLR3* expression in breast cancer and normal tissues were compared, and the results indicated that *TLR3* expression was lower in breast cancer than in the normal controls (Fig. 3A, *p* = 2.52 × 10^–39^). However, no differences in *TLR3* expression were observed among the TNM stages (Fig. 3B, C, and D).

Second, we investigated the composition of tumor-infiltrating immune cells using CIBERSORT. A bar plot was drawn to visualize the proportions of infiltrating immune cells (Fig. 3E). The high *TLR3* expression breast cancer tissues had a high fraction of resting CD4 memory resting T cells (*p* < 0.001), resting Mast cells (*p* < 0.001), and M1 Macrophages (*p* < 0001). The high *TLR3* expression breast cancer tissues had a low fraction of M0 Macrophages, regulatory T cells (*p* < 0.001), and plasma cells (*p* < 0.001).

Next, a Kaplan–Meier plotter revealed that TNBC with high *TLR3* expression had a better prognosis than breast cancer with low *TLR3* expression (Fig. 3F, HR = 0.58, 95% confidence interval [CI]: 0.38–0.88, *p* = 0.0091).

Furthermore, Cox analyses showed that high *TLR3* expression is a protective factor for breast cancer patients (Fig. 3G, hazard ratio [HR] = 0.85, 95% CI: 0.73–0.99, *P* = 0.049). Other risk factors were age (HR = 1.03, *p* = 6.40 × 10^–6^), stage (HR = 2.15, *p* = 1.25 × 10^–10^), tumor size (HR = 1.55, *p* = 6.36 × 10^–5^), lymph node (HR = 1.68, *p* = 3.12 × 10^–8^), and metastasis (HR = 6.22, *p* = 5.20 × 10^–10^).

These results were in keeping with our FUSCC TNBC cohort, wherein lower *TLR3* expression was related to higher breast cancer risk, more advanced stages, and poorer survival rates.

We found similar results in the METABRIC database. We first observed that breast cancer patients with high *TLR3* expression had a better prognosis using the Kaplan–Meier plotter (Fig. S1A, *P* = 0.0015). In addition, we examined *TLR3* levels in both TNBC tissues and normal tissues using the METABRIC database (Fig. S1B), and the result revealed that *TLR3* expression was lower in TNBC tissues than in normal tissues.

### GSEA identifies a TLR3-related signaling pathway

**Fig. 4:**
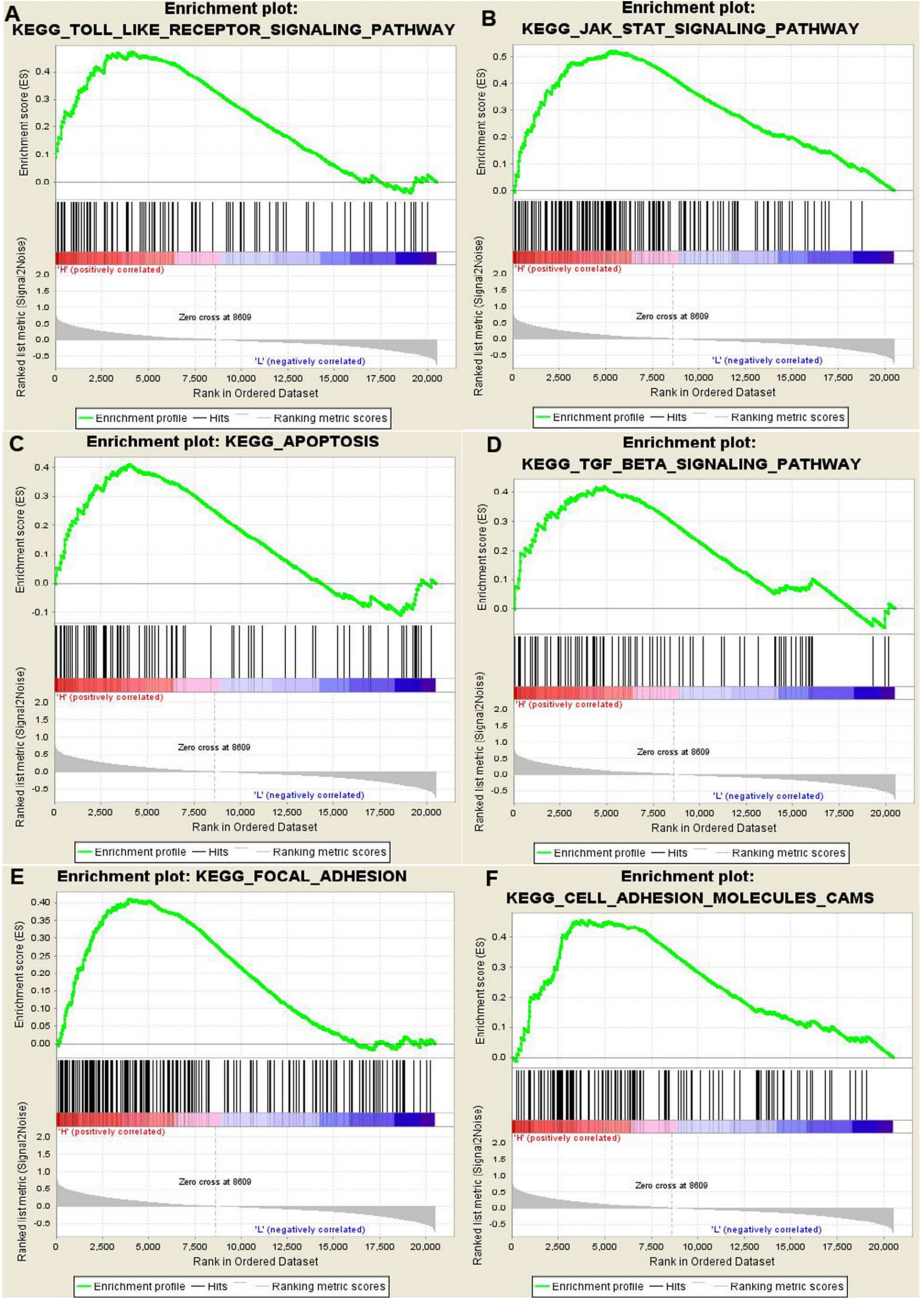
Enrichment plots from gene-set enrichment analysis (GSEA). GSEA showed that Toll-like receptors (**A**), the Janus kinase/signal transducers and activators of transcription (JAK/STAT) pathway (**B**), the transforming growth factor-beta (TGF-beta) pathway (**C**), apoptosis (**D**), focal adhesion (**E**), and cell adhesion molecules (**F**) are differentially enriched in TLR3-related breast cancer

To identify signaling pathways that are differentially activated in breast cancer, we conducted gene set enrichment analysis (GSEA) between low– and high–*TLR3* expression datasets. GSEA revealed significant differences in the enrichment of the MSigDB Collection (c2.cp.biocarta and h.all. v6.1. symbols). We selected the most significantly enriched signaling pathways based on their normalized enrichment score (NES) (Fig. 4). Toll-like receptors, the Janus kinase/signal transducers and activators of transcription (JAK/STAT) pathway, the transforming growth factor–beta (TGF-beta) pathway, apoptosis, focal adhesion, and cell adhesion molecules are differentially enriched in the *TLR3* low-expression phenotype.

## Discussion

In this study, we found that the mRNA expression of *TLR3* was lower in TNBC tissue than in the adjacent normal tissue in the FUSCC datasets. Therefore, we suspect that *TLR3* low expression is associated with TNBC. We performed this study to explore how *TLR3* affects TNBC. We analyzed the expression of *TLR3* in different types of TNBC. The *TLR3* had high expression in IM and MES subtypes and low expression in LAR and BLIS subtypes. To explore the relationship between *TLR3* expression and patient prognosis, we performed a survival analysis. We found high expression of *TLR3* in TNBC predicted better prognosis in the FUSCC TNBC cohort. To verify *TLR3* expression at the protein level, we performed immunohistochemistry on the FUSCC cohort. Immunohistochemical staining of tissue microarrays showed that *TLR3* had lower expression in breast cancer tissues than in the adject normal tissues. Furthermore, We all know that the immune microenvironment of tumors has a very important impact on tumors, so we also investigated the relationship between *TLR3* expression and immune cells, We found that the *TLR3* expression was positively associated with B cell, CD4 + T cells, CD8 + T cells, neutrophils, macrophages, and myeloid dendritic cells. To verify the findings of the FUSCC database, we downloaded additional data from the public databases TCGA and METABRIC for analysis. Bioinformatic analysis using high-throughput RNA-sequencing data from TCGA and METABRIC demonstrated that the reduced expression of *TLR3* in breast cancer was associated with survival time, and poor prognosis.

TLRs are biomolecules expressed on the surface of macrophages, dendritic cells, and epithelial cells that recognize multiple types of pathogen-associated molecular patterns (PAMPs) or damage-associated molecular patterns (DAMPs). TLRs can initiate intracellular signaling, leading to the expression of effector molecules and the secretion of receptors. Recently, many studies have found that aberrant expression and regulation of TLR2,4,7,9 can promote immune escape and angiogenesis of cancer cells [15]. A number of TLRs antagonists are available for the treatment of human cancers. However, TLR3 appears to have a tumor suppressive function.

We previously performed a two-stage association study and found that two SNPs were associated with the breast cancer incidence risk and tumor size. Furthermore, *TLR3* directly inhibited cell proliferation both in vitro and in vivo. TLR3 plays a negative regulatory role in the initiation and progression of human breast cancer cells, at least in part by downregulating the EGFR/PI3K/AKT pathway [12].

Recent studies have suggested that *TLR3* has therapeutic potency in breast cancer. The activation of *TLR3* using a poly (I:C) glycopeptide vaccine induced a pro-inflammatory environment and elicited a strong cellular immune response crucial for breast cancer elimination [16]. Ultimo et al. found that targeting *TLR3* with dsRNA-conjugated mesoporous silica nanoparticles promotes antitumor effects on breast cancer cells [17]. The TLR3 adjuvant, poly-ICLC, plus breast cancer vaccine have been found to provide modest immune stimulation [18]. TLR3 regulates inflammatory factors in the breast cancer microenvironment [19]. However, Schwartz et al. reported that the inhibition of TLR3 signaling using phenyl methimazole (C10) in combination with tamoxifen may increase the effectiveness of the current treatments of breast cancer [20].

To further investigate the functions of *TLR3* in breast cancer, we performed GSEA using TCGA data. GSEA showed that Toll-like receptors, the JAK/STAT pathway, the TGF-beta pathway, apoptosis, focal adhesion, and cell adhesion molecules are differentially enriched in the *TLR3* low-expression phenotype. This finding suggests that *TLR3* may serve as a potential prognostic marker of prognosis and as a therapeutic target in breast cancer. TLR3 is a member of the Toll-like receptors [21]. A recent study found that the JAK-STAT pathway was required for TLR3 signaling to induce synergistic responses for subsequent TLR7 stimulation [22]. *TLR3* stimulation with dsRNA is considered to directly promote tumor-cell apoptosis in many types of cancer, such as breast, melanoma, prostate, cervical, colon, and hepatocellular carcinoma [23–25]. Poly (I:C), a *TLR3* activator, directly causes apoptosis in cancer cells via a caspase-dependent pathway [26, 27].

The results of the gene-set enrichment analysis and the association of *TLR3* expression with clinicopathological characteristics were in accordance with one another. Logistic regression showed that *TLR3* expression was associated with distant metastasis (stage 4 breast cancer). The gene set enrichment analysis suggested that *TLR3* is related to genes that control metastasis, including the TGF-beta pathway, focal adhesion, and cell adhesion molecules. Zagory et al. reported that TLR3 mediates the cell expansion of cells expressing the stem cell marker PROMININ-1 via TGF-beta [28]. Montazeri et al. reported that an interleukin-1 receptor antagonist mediates the Toll-like receptor 3-induced inhibition of cell adhesion [29]. However, most of the above mechanisms were studied in other cells, not in breast cancer cells. The regulatory mechanism needs to be further elucidated in breast cancer cells.

In conclusion, *TLR3* has low expression in TNBC tissue. High expression of *TLR3* in triple-negative breast cancer predicts better prognosis. *TLR3* expression may be a potential prognostic molecular marker of poor survival in breast cancer.

## Supplementary Information

**Additional file 1:** **Figure S1.** Relationship between TLR3 expression and clinicopathological features in the METABRIC cohort. Kaplan-Meier mapping showed that patients with high TLR3 expression in the METABRIC cohort had a better prognosis than breast cancer with low TLR3 expression (A). TLR3 expression in TNBC is lower than in normal tissue (B). TLR3: Toll-like receptor 3. 

## Data Availability

All data needed to evaluate the conclusions in the paper are presented in the paper and/or the Supplementary Materials or can be requested via correspondence upon reasonable request. Microarray data and sequence data from the FUSCC-TNBC cohort have also been deposited in the Sequence Read Archive (WES and RNA-seq; SRA: SRP157974). The datasets used and/or analyzed during the current study are available from the corresponding author on reasonable request.

## Abbreviations

FUSCC: Fudan University Shanghai Cancer Center
TLR3: Toll-like receptor 3
TCGA: The Cancer Genome Atlas
TNBC: Triple-negative breast cancer
TMA: Tissue microarray
PBS: Phosphate-buffered saline
HRP: Horseradish peroxidase
BRCA: Breast invasive carcinoma
TIIC: Tumor-infiltrating immune cells
TNM: The size of the cancer,lymph nodes and metastasis
