# Supplementary figures and images for: High expression of TLR3 in triple-negative breast cancer predicts better prognosis—data from the Fudan University Shanghai Cancer Center cohort and tissue microarrays
Source: BMC Cancer. 2023 Apr 1;23:298. doi: 10.1186/s12885-023-10721-9 (PMC10067281; doi:10.1186/s12885-023-10721-9)

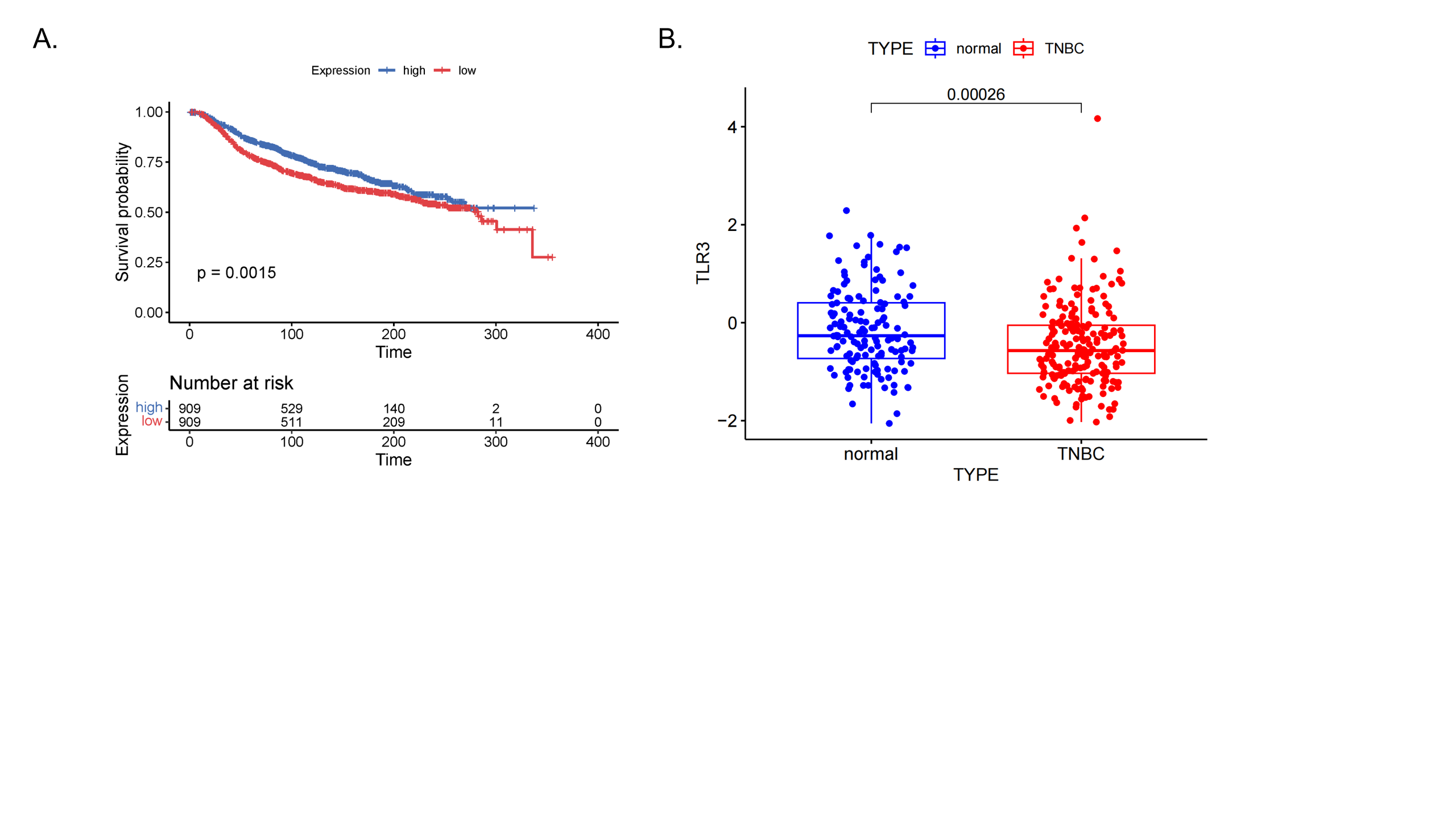

Supplement: Supplementary file 1 — Additional file 1: Figure S1. Relationship between TLR3 expression and clinicopathological features in the METABRIC cohort. Kaplan-Meier mapping showed that patients with high TLR3 expression in the METABRIC cohort had a better prognosis than breast cancer with low TLR3 expression (A). TLR3 expression in TNBC is lower than in normal tissue (B). TLR3: Toll-like receptor 3. [file 12885_2023_10721_MOESM1_ESM.tif]
